# Advances on Bone Substitutes through 3D Bioprinting

**DOI:** 10.3390/ijms21197012

**Published:** 2020-09-23

**Authors:** Tullio Genova, Ilaria Roato, Massimo Carossa, Chiara Motta, Davide Cavagnetto, Federico Mussano

**Affiliations:** 1 Department of Life Sciences and Systems Biology, University of Torino, via Accademia Albertina 13, 10123 Torino, Italy; tullio.genova@unito.it; 2 Department of Surgical Sciences, University of Torino, via Nizza 230, 10126 Torino, Italy; ilaria.roato@unito.it (I.R.); carossamassimo@gmail.com (M.C.); chiara.motta95@gmail.com (C.M.); federico.mussano@unito.it (F.M.); 3 Center for Research and Medical Studies, A.O.U. Città della Salute e della Scienza, 10100 Turin, Italy

**Keywords:** bioprinting, tissue engineering, hydrogels, biocompatible materials, 3D bioprinting, regenerative medicine, orthopedics, dentistry

## Abstract

Reconstruction of bony defects is challenging when conventional grafting methods are used because of their intrinsic limitations (biological cost and/or biological properties). Bone regeneration techniques are rapidly evolving since the introduction of three-dimensional (3D) bioprinting. Bone tissue engineering is a branch of regenerative medicine that aims to find new solutions to treat bone defects, which can be repaired by 3D printed living tissues. Its aim is to overcome the limitations of conventional treatment options by improving osteoinduction and osteoconduction. Several techniques of bone bioprinting have been developed: inkjet, extrusion, and light-based 3D printers are nowadays available. Bioinks, i.e., the printing materials, also presented an evolution over the years. It seems that these new technologies might be extremely promising for bone regeneration. The purpose of the present review is to give a comprehensive summary of the past, the present, and future developments of bone bioprinting and bioinks, focusing the attention on crucial aspects of bone bioprinting such as selecting cell sources and attaining a viable vascularization within the newly printed bone. The main bioprinters currently available on the market and their characteristics have been taken into consideration, as well.

## 1. Introduction

Bone defects are increasing due to bone fractures, osteodegenerative and tumor diseases, thus bone regeneration is necessary to replace the damaged tissue, while the improvement of bone healing, both qualitatively and quantitatively, is mandatory. Every year, approximately a couple of million bone grafts are performed worldwide to treat bone lesions, of which about 1 million only in Europe [1]. Current techniques for repairing bone defects are based on grafting: Autologous grafts (autografts) in 50% of cases (for instance free fibula vascularized grafts); allografts from cadavers or xenograft (bone of animal origin) in 25% of cases; and synthetic grafts (biomaterials as scaffolds) in about 25% of cases [2,3,4,5,6,7,8].

Bone tissue fulfills its functions of withstanding and adapting to mechanical stresses and of fractures healing thanks to a synergy among its components: bone cells, the extracellular matrix (ECM), and bioactive molecules [9]. In addition, a complex cross-talk between bone forming and inflammatory cells is known to guide successful regeneration [10]. Consequently, repairing a tissue in which cells are as carefully coordinated as bone is not an easy task. Only autografts possess all three desirable characteristics of an ideal bone graft: osteoconductivity (i.e., the ability to promote cell adhesion from the recipient site), presence of osteogenic cells from the donor site, and osteoinductive factors. Owing to these premises, autografts are still considered the gold standard for repairing bone defects, although they are not without significant drawbacks, such as donor site availability and possible morbidity. Furthermore, at 10-year follow-up, long-term survival of autologous bone grafts can be lower than 50% [11]. 

Other possible sources (cadaveric allografts and animal xenografts) avoid donor site morbidity, but present poorer biological properties, such as lower biocompatibility, a more difficult graft integration, and a risk of viral or bacterial infection. Synthetic bone grafts and biomaterials mainly show osteoconductive properties, they can be degraded by osteoclasts, then substituted simulating the physiological remodeling, but they are most suitable for small defects [11]. 

To overcome the pitfalls of the current procedures mentioned above, researchers have oriented their endeavors to bone tissue engineering (BTE), a branch of regenerative medicine (RM), enabling the production of cell-laden scaffolds, in which bone biological components are assembled to form a three-dimensional (3D) environment [12,13,14]. This innovative avenue of research, harbinger of ground-breaking therapeutic options, has been recently boosted by the advent of a series of techniques, commonly defined as bioprinting, that allow to repair bone defects through 3D-printed living tissues [15]. 

An even more compelling point in favor of 3D-bioprinted constructs is represented by the attainment of biomimicry and hence the possibility of avoiding an abnormal immune reaction towards grafts, the well-known foreign body reaction, which may lead to chronic inflammation, fibrosis, or scarring and transplantation failure [16,17]. Indeed, according to the different physical, chemical, and biological properties, the various scaffolds used for bone implants can exhibit different immune responses. On the other hand, immune cells control osteoclastogenesis, osteogenesis, and the process of bone healing through the release of regulatory factors [18].

Bioprinting technology deeply improved the availability of effective synthetic-bone substitutes with enhanced performance, in the last years. This review discusses the main factors that are critical for bioprinting in BTE.

## 2. Bioprinting 

3D bioprinting is a cutting-edge technology with a broad utility in BTE and RM [19,20]. It is used to build constructs starting from a single cell type using layer-by-layer deposition of specific bioinks, which are essentially the biological components needed for the scaffold. Therefore, 3D bioprinting allows to develop highly reproducible, spatially controlled structures made of different materials, growth factors and cells, such as synthetic bone substitutes. 

The great advantage of 3D bioprinting relies in the potentiality to spatially distribute the cells within the solid or semi-solid biomaterials, thus optimizing tissue regeneration [21]. The development of 3D-bioprinted bone tissues is of great relevance and impact on clinical practice, because it also allows the reconstruction of bone defects with complex shape, just by translating computed tomography (CT) or microCT data of defects to printable image of them, leading to patient-specific implants [22,23,24]. The ideal scaffold should resemble a 3D structure and composition of human bone, it has a resorption rate that gives time to the bone from the recipient site to replace it, it provides nourishment of the graft cells and allows vascularization, which is essential for the graft success and a higher bone healing ability compared to non-osteoinductive ceramics [25].

Moreover, 3D bioprinting allows the production of constructs with different geometrics, porosity, and sizes, which are features relevant to obtain more osteoinductive scaffolds. Osteoinduction is a fundamental process, thereby osteogenesis is induced; it implies the commitment of undifferentiated progenitor cells towards osteoblasts/osteocytes. An effective osteoinduction is achieved after heterotopic implantation induced by bone morphogenetic proteins (BMPs) [26]. Generally, osteoblasts or progenitor cells need a proper stimulation by BMPs for osteogenic differentiation, but some biomaterials can induce an intrinsic osteoinduction, where mesenchymal stem cells (MSCs) differentiate into osteoblasts, even without exogeneous BMPs, avoiding the adverse effects of BMPs treatment. Indeed, in patients, the use of high BMP doses has been associated with numerous serious adverse effects, such as ectopic bone formation with spinal cord compression [27], increased bone resorption due to a transient elevated osteoclast activity [28], life-threatening cervical swelling [29], and structurally abnormal and mechanically unstable bone tissue formation, currently limiting the overall clinical efficacy of BMPs [30]. To overcome the negative effect of BMPs, research efforts have been performed to identify and utilize materials with intrinsic osteoinductive properties. For instance, in vivo studies have demonstrated that some calcium phosphate (CaP) ceramics may present intrinsic osteoinductive properties, when implanted ectopically [26].

### 2.1. Considerations on Bioinks and Scaffold Nature for Additive Manufacturing

The success of bioprinting and of printed constructs are related to different factors pertaining to the bioinks and the nature of the scaffold surface. Bioinks useful to obtain effective bone substitutes require properties including biocompatibility, biomimicry, biodegradability, bioprintability, and mechanical integrity [31,32]. Thus, the design of the appropriate bioink is probably the main challenge of bioprinting [33,34]. For instance, parameters such as bioink viscosity, the effects of pressure, temperature, nozzle size, crosslinking methods on bioinks, and the macrostructure/geometry of the material (i.e., porosity) are major concerns for the successful production of bone tissue [35,36]. 

Bioinks are the key components of bioprinting technology; they include printable organic and inorganic materials, biological factors, and other components that enhance cell growth, differentiation, and preserve shape fidelity during free-form deposition as extruded filaments [37,38,39,40]. Depending on the final aim, the cells can be deposited onto the scaffold biomaterial during the printing process, generating the scaffold-based bioinks [33] or, alternatively, they can be directly printed embedded in the biomaterial, implementing the scaffold-free bioinks [41,42,43]. 

Importantly, bioprinting of bone requires the use of bioinks capable of transitioning from a liquid state to a gel structure, without compromising cell viability and bioactivity [24]. Since bone is exposed to different and not uniform mechanical stress, and to various nutritional and vascular needs, bioinks must possess physical properties providing aid for cell differentiation by ensuring a favorable 3D microenvironment [44]. Starting from the introduction of cross-linkable bioinks, such as methacrylated gelatin and hyaluronan, more and more new materials are being engaged to make optimized bioinks. Another approach relies on the use of composite materials, which combine the advantages of each bioink, improving their mechanical strength, printability, biocompatibility, and gelation characteristics [45,46,47]. 

Macrostructural and geometry properties of material have a deep impact on the effectiveness of a scaffold, because porous materials, characterized by numerous pores of variable size and connectivity, are suitable for the passage of oxygen, nutrients, and cellular wastes. Notably, the porosity of the cell-laden scaffold is known to affect tissue formation and concomitant angiogenesis, which are two critical aspects for BTE [48,49,50]. Tarafder et al. [51] showed in a rat model that the control of the pore size resulted in an increased compressive strength, cell density, biocompatibility, and osteogenesis [51]. Various scaffolds based on different bioactive nanomaterials have been tested for their capabilities to induce new bone formation. For instance, hydroxyapatite (HA) nanoparticles showed a favorable osteoinductive activity on MSCs. In particular, HA nanostructured with concave macroporosity, derived from CaP crystals, accelerate osteoinduction, since they are chemically and structurally similar to those of the natural bone tissue [52]. Other nanoparticles made by different components, such as molybdenum-doped bioactive glass [53], magnetic iron oxide [54], strontium containing bioactive glass [55], and gold [56] showed osteoinductive abilities. 

### 2.2. Biomaterials 

Biomaterials currently used in 3D-bioprinting can be mainly classified as: Non-hygroscopic polymers, hydrogels, and decellularized extracellular matrix (dECM) [57].

#### 2.2.1. Non-Hygroscopic Polymers

**Figure 1:**
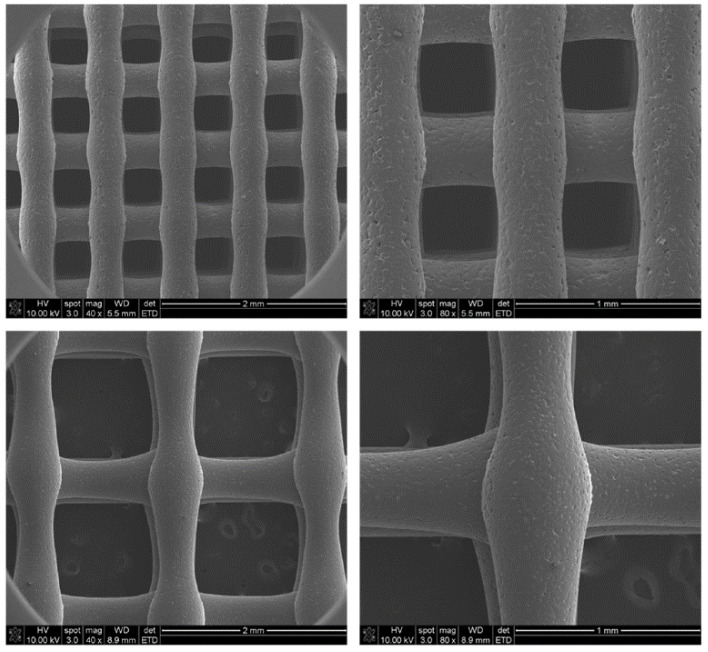
Characterization of scaffold using SEM analysis. SEM images of polycaprolactone (PCL) scaffolds (upper: 400/400 scaffold, lower: 400/1200 scaffold) (original magnification: Left, ×40; Right, ×80). Picture taken from Park et al. [59] under the terms and conditions of the Creative Commons Attribution (CC BY) license https://creativecommons.org/licenses/by/4.0/.

Synthetic polymers can be synthetized with controllable chemical, physical, and biological properties. They are, in general, mechanically robust and durable and can be used as structural support in tissue engineering (TE) (Figure 1) [58]. 

Synthetic polymers show low immunogenicity, but have also disadvantages since they lack biological cues, thus they do not stimulate cell and tissue-material signal interactions. Moreover, they are less biocompatible than other options, due to the required use of cytotoxic solvents or high temperatures necessary for printing them. Their biodegradation after implantation produces lactic acid and carbon dioxide, which create an acid environment that favors inflammation instead of healing [60]. 

The most widely utilized polymers for BTE are polycaprolactone (PCL), thermoresponsive biodegradable polyurethane (PU), Poly-Lactic Acid (PLA) and Poly-lactic-co-glycolic acid (PLGA). 

PCL shows slow biodegradation rate, it has good mechanical properties, it favors cell adhesion and proliferation [61,62]. Aural cartilage reconstruction and mandible bone regeneration were achieved using printed PCL as supporting scaffold. PCL presents a lower melting point than other melt-cure materials, which reflects a reduced temperature-induced cell damage [63]. Until recently, PU foams, although widely used in surgical training, were unsuitable for customized 3D printing. Owing to a few pivotal studies, this limit seems to have been overcome. In 2015, PU was successfully printed as a structural support for cell laden bioinks [64]. Even more compelling, for the first time in 2019, different piperazine (PP)-based polyurethane-urea (P-PUU) scaffolds were fabricated [65], through 3D printing technology. These materials showed a suitable interconnected pore structure supporting excellent biocompatibility and osteoconductivity, as assessed in vitro and in vivo. 

Almost chemically and biologically inert, PLA is a hydrophobic polymer endowed with remarkable mechanical strength, thermal stability, and suitable biodegradable features [66]. When used in vivo, it was shown to directly degrade by hydrolysis without the use of catalysts or enzymes [38].

PLGA is biocompatible but shows a poor osteoconductivity [67]. It has a monitored biodegradation rate since its degraded products (lactic acid and glycolic acid) can decrease the pH of surrounding tissue, stimulating an immune response [68]. Sawkins and colleagues [69] successfully reproduced the properties of human trabecular bone in terms of elastic modulus and yield point by printing PLGA-based constructs.

Compared to melt-cured polymers, photo-cured ones are easier to print through the layer by layer deposition technique and they show higher mechanical integrity [38,70]. These advantages, however, are, at least in part, counterbalanced by the possible cytotoxicity of the free radicals produced during the polymerization process [71]. Notwithstanding the huge amount of research performed so far, novel polymers and resin components may be needed, along with a more systematic approach to process optimization. Moving toward this direction, Guerra et al. [72] investigated the effects of poly(propylene fumarate) resin components on 3D printing process parameters, with a particular emphasis for the methodological soundness.

#### 2.2.2. Hydrogels and Composite Scaffolds

**Figure 2:**
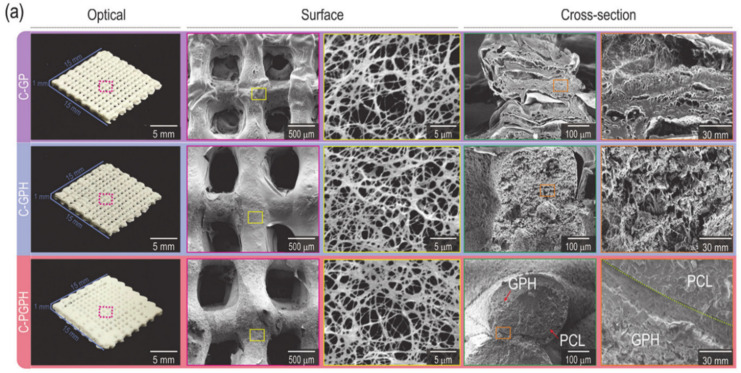
Optical and SEM images of C-GP, C-GPH, and C-PGPH scaffolds. Picture taken from Kim et al. [73] under the terms and conditions of the Creative Commons Attribution (CC BY) license https://creativecommons.org/licenses/by/4.0/.

Hydrogels are polymers capable of absorbing and retaining great quantities of water [33]. They are the most common scaffold-based bioink (Figure 2). 

Cell-laden hydrogels support cell proliferation and growth, enhancing the formation of 3D tissue constructs. Contrary to other polymeric scaffolds, in which cells are generally seeded on the surface of scaffolds, hydrogels provide a 3D environment, where embedded cells can migrate freely and interact with each other within the porous flexible network [74,75,76].

As for their source, hydrogels are classified into natively and synthetically derived hydrogels. Naturally derived hydrogels resemble the native tissue environment since they provide essential features of the ECM components [77], such as a hydrated and mechanically strong 3D microenvironment, where cells can be encapsulated. Among the most common hydrogels of natural origin used in 3D bioprinting there are hyaluronic acid (HyA), collagen, agarose, chitosan, fibrin, alginate and Matrigel. 

Compared to the abovementioned hydrogels, synthetically derived hydrogels have the advantage to allow easy modification of their mechanical features and cell-adhesion properties. Methacrylated gelatin (GelMA), polyethene glycol (PEG) and Pluronic F-127 belong to this category. Owing to the presence of specific photoinitiators, GelMA and PEG are both photocrosslinkable, when exposed to specific wavelengths [33]. GelMA is widely used in light-based bioprinting (LBB) and extrusion-based bioprinting (EBB) [78].

With regards to their polymerization process, hydrogels may be either thermoresponsive or photocured. Among the most diffused thermoresponsive hydrogels for 3D bioprinting [79,80] there are Pluronics, agarose, Matrigel, and gelatin. Pluronic hydrogels possess good printability, however poor mechanical features and reduced degradability have hindered their application [81]. On the other hand, agarose and Matrigel, albeit mechanically stronger than the Pluronic gel, are affected by unsatisfactory printability and poor resolution [82]. Gelatin is partially denatured collagen, it is biodegradable and highly biocompatible, but it cannot remain in the hydrogel state at the body temperature, gelling below 28 °C; thus, adding a cross-linking moiety becomes mandatory [83,84]. 

Photo-curing hydrogels are among the most promising materials. In 2015, Gao et al. [85] designed a bioink with adequate mechanical properties to create bone and cartilage substitutes. This bioink was composed of MSCs and PEG-gelatin cross-linked with GelMA hydrogel. Twenty-one days after printing, it showed the improvement of elastic modulus of human MSC-PEG-GelMA constructs by 100% compared to the cell laden PEG or GelMA constructs. The findings of the study demonstrated that photo-cross-linkable multimaterial hydrogels are promising bioinks to create bone and cartilage substitutes.

Bioactivity and printability are paramount aspects to be considered for hydrogel-based bioprinting. Indeed, higher concentration and crosslinking density normally favor a better printability and shape fidelity, but a smaller pore size and lower cell viability. Thus, low concentrated hydrogels are utilized mainly as cell-encapsulation materials for bioprinting [86]. To obtain hydrogels with both bioactivity and printability, a composite material constituted by low-concentration GelMA and gelatin was created, which resulted as comparable to highly concentrated hydrogel [33]. In 2017, Bendtsen et al. [87] proposed a novel hydrogel composed of alginate, polyvinyl alcohol (PVA), and HA showing excellent rheological properties and high shape fidelity, with optimal printability and biocompatibility. Furthermore, its osteoconductivity made it a favorable environment for new bone formation. Hsieh et al. [88] developed a PU-gelatin composite bioink that allowed high-resolution printing, long working windows, tunable mechanical properties, and degradation rates providing a conducive microenvironment for cell growth.

#### 2.2.3. Decellularized Extracellular Matrix_Based Bioinks

**Figure 3:**
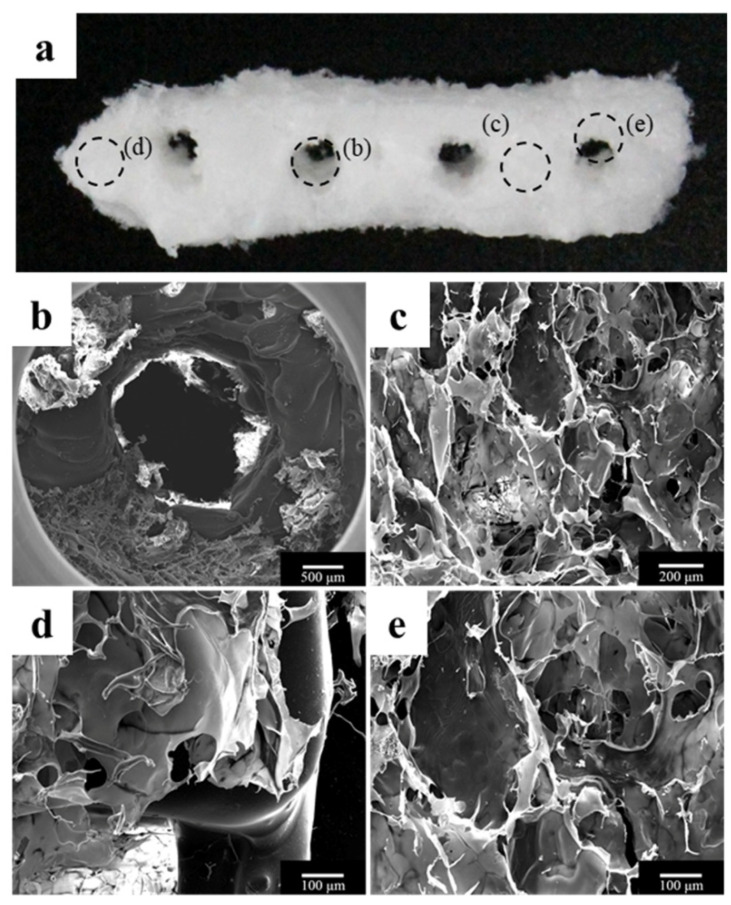
Morphology of the polycaprolactone (PCL)/β-tricalcium phosphate (β-TCP)/bone decellularized extracellular matrix (dECM) scaffold. (**a**) Visual image of the scaffold; (**b**) an implant through hole, which plays a role in guiding the implant fixture; and (**c**–**e**) bone dECM coated on the scaffold. Picture taken from Bae et al. [89] under the terms and conditions of the Creative Commons Attribution (CC BY) license https://creativecommons.org/licenses/by/4.0/.

Usually, once printed, cells start to self-assemble and to form functional tissues in the native-like ECM. Indeed, scaffold mimics temporary the ECM and allows cells to work and create the real ECM, then substitute it (Figure 3). 

The ECM in bone tissue mainly consists of Collagen Type I (about 95%), which display a D-banding ultra-structure due to self-assembly of collagen triple helices, thus conferring mechanical strength to the bone [90]. In addition, other non-collagen proteins (i.e., alkaline phosphatase, osteopontin, fibronectin, osteonectin) are involved in the formation of ossified ECM, HA nucleation, and growth. A possible strategy to produce bone tissue in vitro is to employ natural dECM to overcome the problem of immunogenicity and to better resemble the native environment. dECM is a biomaterial that retains the native ECM components. To be prepared, dECM bioinks require the removal of cells from a tissue (through chemical, physical, and enzymatic processes) while preserving only the ECM [91]. DECM is then solubilized to a desired concentration to give rise to a gel-like substance that is appropriate for 3D bioprinting. DECM is an excellent allogenic or xenogeneic biomaterial for tissue engineering and yielded different commercial products such as Alloderm^®^, SurgiSIS^®^ and Synergraft^®^ [92].

Once isolated, the bone dECM can be co-printed with biocompatible hydrogels [93,94]. Furthermore, different scaffold materials have been developed trying to reproduce the ultra-structure of native bone ECM [95]. To promote osteogenesis, small particles of HA or β-TCP can be dispersed in the hydrogel-based bioink. Indeed, the nanotopography (in the range of 60–80 nm) of the scaffold strongly influences cell behavior, better resembling native ECM [96,97,98]. In particular, nano-sized particles enhance HA deposition and they release ions that promote stem cell differentiation (osteoinduction) [99,100,101,102]. Hence, to improve the process of osteo-differentiation, multiple geometries of 3D-printed HA have been tested [103]. Moreover, the osteogenic ability of HA/PCL conjugates have been investigated in femur and lumbar spine of rabbit [104]. HA particles bioprinting has been applied in vivo for in situ bioprinting purposes. In this case, HA nanoparticles deposition was performed directly into defective mouse calvaria by using a laser-based bioprinting system [105,106].

Moreover, dECM material has proved effective in bone regeneration as indicated by the increased expression of osteogenic genes by human adipose-derived stem cells (ASCs) seeded on dECM-PCL scaffolds compared with those maintained in PCL scaffolds [107]. The advantage of incorporating dECM with respect to hydrogels alone is due to its unique composition able to provide the right environment necessary to incorporate cells. Hydrogels cannot resemble the complexity of the natural ECM microenvironment. dECM presents some disadvantages compared to hydrogels: reduced post-printing shape maintenance and ethical issues due to its derivation. Indeed, human-derived ECM is the ideal source for implantation since xenogenic dECM can stimulate an immune response [108]. 

### 2.3. Cell Sources

Cell sources currently used in 3D bioprinting are primary cells or stem cells [106]. The cells are deposed in predetermined patterns to produce tissue constructs that well resemble their native counterparts. The perspective to utilize adult stem cells, such as MSCs, to create 3D tissues for RM is tempting since it opens up the availability to individualized, patient-specific stem-cell-based treatments. Indeed, the ability of adult stem cells to differentiate into specific cell types can facilitate the fabrication of tissue-specific implants. MSCs showed a positive biosafety profile [109] because they can be cultured for weeks without adverse consequences, thus they are currently in clinical trials with encouraging results. However, adult stem cells are scattered throughout tissues and their expansion is impaired or limited by their proliferative ability. To overcome these limitations, a solution is represented by induced pluripotent stem cells (iPSCs), which have been tested in many well-reviewed studies [110,111]. IPSCs can be produced from different cell types, but most often from fibroblasts. One challenge of printing iPSCs is their tumorigenic potential, which has not been definitively investigated. IPSCs are produced in a way similar to a tumor formation assay called “focus formation” in fibroblasts, that is a monolayer culture of fibroblasts, transduced with retroviruses, which form colonies at high density without passaging. These colonies exhibit escape from the normal quiescent state induced by contact inhibition, thus iPSCs foci are transferable to form new cultures and can cause tumors when injected into immunocompromised mice [112]. A more recent work by Nguyen et al. [113] showed that iPSCs maintain a pluripotent phenotype after 3D bioprinting with different bioinks, and that the expression of genes associated to tumorigenicity was undetectable in the prints after five weeks of differentiation, suggesting a non-tumorigenic behavior. 

Keeping in mind the above described points, MSCs result as the most used cells in this field, and MSCs derived from bone marrow [114] and adipose tissues [115] especially have been used in bioprinting artificial tissues/organs [85,116]. Human ASCs have been shown to upregulate osteogenic genes when bioprinted with decellularized bone (DCB) matrix and PCL as bioinks [107], consistently with their known capability to differentiate into osteoblasts [117,118,119,120]. Furthermore, adult stem cells differentiation toward bone lineage is enhanced by BMP-2 [121]. In another work, PCL filaments were utilized as support for collagen or HA hydrogel networks, containing MSCs blended with BMP-2 or transforming growth factor beta (TGF-β) for bone or cartilage engineering, respectively [122].

### 2.4. The Importance of Rheology in Bioprinting

Rheology is the study of flow properties of materials under external forces. The rheology of a given biomaterial ought to be considered in order to ensure cell viability within the ink [123]. Properties such as printability [124,125] are typically affected by shear stress and thus depend on rheology [126]. Blaeser et al. [127] investigated the effects of bioink compositions and printing pressures on human MSCs’ viability, showing that post-printing cell viability significantly decreased by increasing shear stress. Ouyang et al. [128] systematically investigated the rheological characteristics of a gelatin/alginate mixture with gelatin as a major component for gel formation for bioplotting Embryonic Stem Cells, under different parameter combinations. They showed that both printability and viability are influenced by printing temperature, gelatin concentration, and holding time. Moreover, Aguado et al. [129] reported that the higher is the viscosity, the greater the vitality is.

The level of shear stress is directly influenced by different printing parameters (Table 1), such as nozzle diameter, printing pressure, and viscosity of the dispensing medium [130,131,132,133]. For instance, the wall of the nozzle tip and other areas of the printer induce a shear stress, reducing cell viability, and modifying the fluid properties [134]. In another work, Muller et al. [135] created an algorithm to compute full velocity, shear rate, and viscosity profile in a printing nozzle for generalized Newtonian fluids such as shear thinning bioinks. Geometric constraints of the printing apparatus (needle shape and size) can influence shear stress; indeed, large-orifice deposition needles reduce it, but also 3D print resolution and lower volumetric flow rates decrease the shear stress [134]. 

### 2.5. The Importance of Vascularization

Vascularization is a critical component for bioprinted tissue [137], and it is still a major issue in bioprinting both in BTE and RM. Vascular network incorporation should always be considered in the construct production process, thus providing oxygen, nutrients, and avoiding tissue death [138,139]. Since angiogenesis and osteogenesis are highly inter-connected processes, the presence of a functional vascular network is particularly relevant to produce bone grafts. 

3D-printed bone substitutes, characterized from a poor vascularization, could result in the failure of the constructs after implantation, above all in large bone defects. Recent technological advancements in bioprinting allowed for printing endothelial cells together with the other cellular and non-cellular components, recreating complex vascularized structures. Temple et al. [140] produced PCL scaffolds with different porosity, according to the shape of human mandibular and maxillary bones, which were colonized by ASCs and resulted in an effective vascularized bone formation. Byambaa et al. [141] developed a novel hydrogel that allows a co-culture of bone marrow derived human MSCs and human umbilical vein endothelial cells (HUVEC), to provide functional vasculature in large bone defects. The GelMA hydrogel conjugated by vascular endothelial growth factor (VEGF) and loaded with silicate nanoplatelets promoted simultaneous angiogenesis and osteogenesis. Three weeks after in vitro culture, the constructs showed high cell viability, proliferation rate, and structural stability. The results also indicated the formation of a mature bone niche after 21 days of culture [141]. Moreover, Lv and colleagues [142] proved that a prolonged release of VEGF through 3D bioprinting improves both osteogenesis and angiogenesis. Anada et al. [143] obtained a highly vascularized biomimetic hydrogel suitable for BTE applications, by creating a dual ring bone-mimetic construct, composed of a GelMA/octacalcium phosphate in the external ring, which stimulated bone formation, while the central ring of GelMA loaded with HUVECs promoted angiogenesis within the construct. Fedorovich et al. [144] described how to effectively bioprint vascularized bone tissue by using alginate hydrogels and Matrigel^TM^, seeded with endothelial cell precursors and MSCs, thus creating a heterocellular and multimaterial construct. The derived grafts were tested by subcutaneous implantation in an animal model (immune-deficient mouse). The presence of osteoinductive materials (CaP ceramics) and growth factors embedded in the construct, both determinant for stem cells differentiation, promoted MSCs differentiation into bone forming cells and ectopic bone deposition after 6 weeks.

Poldervaart et al. [145] tested Matrigel in combination with alginate (which improves printability) as a bioink for vascularization studies, also incorporating VEGF into the bioink either directly or within microspheres, which enabled its controlled release. They showed that, although alginate improved bioprinting, the degradation rate increased, while the rate of formation of vessel-like structures decreased. Several methods can be adopted to create vasculature, for instance, small channels can be printed using EBB in combination with fugitive inks and later on, they can be populated with endothelial cells to reproduce vessel-like structures [146,147]. Alternatively, channels lined with endothelial cells can be directly produced using bioprinting. For instance, Dolati et al. [148] described a system with coaxial nozzles capable of printing perfusable vascular ducts. A further improvement in the production of bone tissue with blood vessels could be represented by 3D-bioprinted organ-on-a chip platforms, where 3D artificial tissue is directly printed within microfluidic devices [149]. Indeed, 3D printing enables to produce microfluidic device with a specific architecture, showing capability to control fluid and physical features spatially and temporally. These devices allow to study complex biological mechanisms, such as bone angiogenesis; indeed, Jusoh et al. [150] created an in vitro model of vascularized bone tissue, by developing a fibrin and HA-based vascular network within a matrix.

## 3. Bioprinting Process

To create complex and vital structures, correct management of pre- and post-printing operations is particularly relevant. The pre-printing operations concern a correct design and planning of the structure needed according to the function that this structure should have in vivo, taking into account any different operating temperatures and appropriate printing times in which to insert the cells (or different kind of cells).

To obtain anatomically correct tissues/organs, the use of computer-aided design/computer-aided manufacturing (CAD/CAM) technologies, in combination with bioprinting, has proven extremely helpful [151,152]. 3D printers assure the manipulation of the bioinks at high resolution and following specific designs [31,139,153].

A fundamental step for the transition to clinical application is the development of integrated systems that put together 3D-bioprinted constructs and bioreactors [154,155]. Post-printing operations are essential to keep these structures viable; indeed, large and complex structures cannot be treated as normal cell cultures, but require active support to satisfy their metabolic and stimulatory needs.

Bioreactors allow the development of a proper microenvironment that is essential to produce constructs that fully resemble the native tissue. This is especially true for bone tissue, which needs a stepwise increase in mechanical stress while proceeding through differentiation [156]. Most bioreactors show low volume of output, thus requiring a lot of time for tissue formation. If bioreactors will become able to closer mimic real body conditions, the cell growth and differentiation will improve as well as the success rate of 3D bioprinted tissues. 

**Figure 4:**
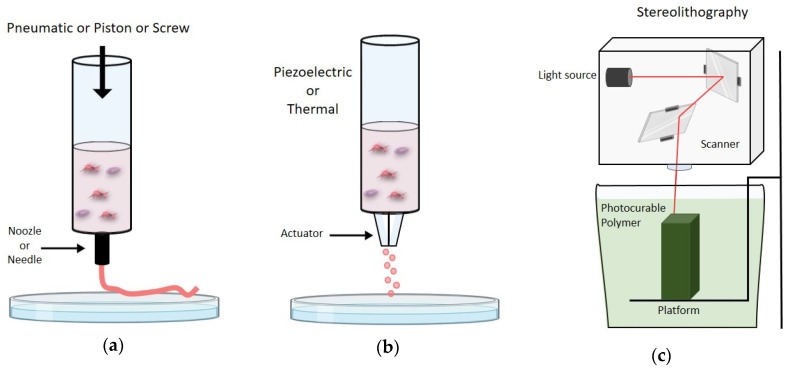
(**a**) Schematic representation of extrusion based bioprinting; (**b**) schematic representation of ink-jet based bioprinting; (**c**) schematic representation of light based bioprinting.

The production of 3D-bioprinted structures is based on three essential modalities (Figure 4), which can be used alone or in combination, named extrusion-based, inkjet-based, and light-based (or laser-based) bioprinting [123].

One of the most used mechanism of hydrogel bioprinting is the layer-by-layer deposition and crosslinking scheme. This feature makes hydrogels’ bioprintability superior to that of other bioink types [33]. 

### 3.1. Extrusion-Based Bioprinting

Extrusion-Based Bioprinting (EBB) deposits cells and biomaterials to a substrate by a direct contact using a syringe through a cylindrical extrusion process, which can be pneumatic or mechanical [157]. Piston-driven systems ensure direct control on the speed of bioink deposition, which in pneumatic-based systems may undergo delays associated with the compressed gas volume. On the other hand, screw-based deposition is more suitable for highly viscous bioinks and provides better spatial control [158].

EBB has relatively poor resolution, with 100 um as the optimal [159,160], but it is particularly suitable for bioinks with high viscosities and high cell densities [31]. This feature enables the production of 3D bioprinted constructs that better resemble the cell density of the native tissue. Other strengths of this technique are the high structural integrity due to continuous filaments deposition and the wide range of speeds [57]. EBB has the greatest flexibility among existing bioprinting modalities, due to the extrusion mechanism as well as the larger nozzle diameters. Hence, it can be used in association with a wide range of bioinks, including both scaffold-free and scaffold-based (e.g., hydrogels) inks. Additionally, this technique allows for preserving cell viability (40–80% post-printing viability is usually observed) [33,132,139] and, by using multi-channel printing systems, to obtain high levels of structural and functional complexities, such as cartilages and bones [42,63,161].

### 3.2. Inkjet-Based Bioprinting

Inkjet-based bioprinting (IBB), also known as droplet-based bioprinting (DBB), enables the formation of tissue constructs by releasing the bioink in the form of liquid droplets [162]. This technique has higher resolution than EBB and it is superior to EBB to generate micro-tissues, which are sub-millimeter constructs able to mimic the structures and functions of native tissues [163]. By exploiting fluid properties such as surface tension and viscosity, the 3D structure takes shape [162,164]. Four different methods are currently used to form bioink droplets: inkjet, electro-hydrodynamic jet, acoustic-droplet-ejection, and micro-valve [165,166,167,168,169,170]. Since droplets curing following ejection is quite slow, the resolution in *Z*-axis cannot be elevated. DBB presents several advantages such as the high printing speed and relative low costs [162]. The main applications of DBB technology regard skin [171], cartilage [172], bone [173,174] and blood vessels bioprinting [34,175,176]. Bone-like structures have been produced using the IBB technology, which allows HA and tricalcium phosphate droplets deposition onto powders [34,173,174].

Using thermal-IBB Gao et al. [34] produced poly(ethylene glycol) dimethacrylate scaffolds supplemented with HA and osteoinductive ceramics co-printed with bone-marrow-derived human mesenchymal stem cells (BMSCs). This technique presented significant advantages compared to manually pipetted BMSCs, such as an even and 3D homogeneous BMSCs distribution. After three weeks of in vitro culture, it presented better cell viability, collagen production and alkaline phosphate activity. Due to the underlie mechanism of biomaterial deposition, DBB is used in association with bioinks characterized by low-viscosity (3.5–12 mPas) and low cell density. Moreover, cell viability can be affected when high pressures are adopted. This limits DBB capability in recreating tissues composed of cells at high concentrations; moreover, considering the rheological properties needed for this process, it appears evident that the range of suitable bioinks is limited compared to EBB. Recently, to overcome the problems of DBB, a novel direct-volumetric drop-on-demand (DVDOD) technology has been developed, resulting in the generation of functional tissues. This technique allows for dispensing bioinks with highly concentrated cells and viscosity biomaterials [177]. 

### 3.3. Light-Based Bioprinting

The third method for 3D-bioprinting is Light-Based Bioprinting (LBB), also commonly referred to as laser-based bioprinting. LBB, which includes laser-assisted printing and stereolithography, configures as the fastest and most resolute method among all bioprinting strategies, with no limitations associated with the material viscosity [34,178,179]. 

LBB utilizes a light pulse directed via mirrors onto a bioink layer above the substrate. In processes based on stereolithography, the final construct is obtained by repeated cycles of photopolymerization of the liquid biomaterial. On the other hand, processes based on cell transfer (i.e., laser-induced forward transfer) [180,181] do not harm the printed cells, which maintain their viability in a very high percentage with a minimal expression of heat shock proteins [180,181]. The basic set up of this technology consists of two coplanar glass slides, where cells are suspended after proper manipulation in a medium with adequate viscosity, usually a hydrogel. The upper glass slide, i.e., donor-slide, carries underneath the layer to be transferred. At a distance ranging from 10^−4^ m to 10^−2^ m, the collect side, endowed with an absorbing layer, receives the material while it is processed avoiding dehydration and cushioning the impact. 

Among the different light sources used for LBB, laser is the best known; nevertheless, UV lamp and light-emitting diode (LED) sources are widely used too. Importantly, the use of UV radiation might cause oxidative cell damage and death by promoting free-radicals production [182]. 

Compared to EBB and DBB, LBB technology displays a higher complexity, especially in terms of process control and machinery. All these variables could increase the risk of cell damage and biomaterial deterioration [183]. 

Moreover, due to the requirement of bioinks needing specific criteria of fluid mechanics or cross-linking speed, the versatility of LBB is lower than DBB [181].

Multi-nozzle systems for LBB have been recently developed. For instance, Kang and colleagues validated a four-cartridge system, named integrated tissue-organ printer (ITOP), to engineer mandible and calvaria bone, cartilage, and skeletal muscle [63]. 

This method enables the production of 3D structures in microfluidic devices, well resembling the native microenvironment [184,185].

### 3.4. 3D Bioprinting Applications to Treat Bone Defects

Besides BTE, 3D bioprinting is strongly relevant in the field of cancer research, where 2D tumor models do not reconstitute the complexity of the dynamic tumor microenvironment [186]. Conversely, 3D-bioprinted models allow for reproduction of cell–cell and cell–matrix interactions and have the advantage to integrate a vascular system to study tumor angiogenesis [187]. Hence, the tumor tissue should be placed within a bioprinted vascularized parenchyma to analyze how cancer cells grow and other carcinogenic events, i.e., intravasation and extravasation [188]. Important to note is that a 3D biomimetic bone matrix has been used to create a model of breast cancer bone metastases, with a bone like microenvironment that provides cross-talk among breast cancer cells, human bone marrow MSCs, and osteoblasts [189]. Zhu et al. [190] used a 3D printed nano-ink, made of hydroxyapatite nanoparticles suspended in hydrogel, to simulate a bone-specific environment to study breast cancer bone invasion. 

The potential applications of BTE in orthopedics are enormous since can solve both bone and cartilage problems [191]. A comprehensive review analyzing the application of BTE for orthopedic trauma according to the different anatomical sites, showed its usefulness to treat bone trauma in a patient-specific manner [192]. Alba et al. [193] developed a new method to engineer periosteum tissue by printing periosteal derived cells (PDCs) mixed with alginate on collagen scaffolds. The presence of collagen contributed to maintain the structural integrity and osteogenic differentiation of PDCs, which was demonstrated by osteocalcin and alkaline phosphatase gene expression. 

A multi-component bioink, constituted by wood-based nano-cellulose and bioactive glass to strengthen gelatin-alginate bioinks, was tested and resulted effective in sustaining bone cell viability, proliferation, and osteodifferentiation [193].

Cartilage tissue defects are difficult to repair due to cartilage poor self-repairing capacity, thus the potential to re-create functional articular cartilage by 3D bioprinting is contemporary tempting and challenging. Cartilage must sustain heavy loads, therefore a hybrid scaffold, constituted by PCL with rabbit chondrocytes and fibrin collagen hydrogel, was fabricated to enhance mechanical and biological properties for load-bearing cartilage. The authors showed that this hybrid construct formed cartilage-like tissues both in vitro and in vivo, as evidenced by the deposition of type II collagen and glycosaminoglycans [194]. Daly et al. [195] used an MSC-laden bioink (arginine-glycine-aspartic acid (RGD)-modified alginate hydrogels) co-deposited with PCL fibers, which showed s 350-fold increase in compressive modulus of bioink/PCL templates. The constructs had the potential to be implanted as vertebral bodies in load bearing locations.

O’Connell et al. [196,197] developed a device named “Biopen”, which is basically an EBB bioprinter for in vivo application directly during the surgery. This Biopen was utilized to repair chondral defects in a large animal ovine model [198]. Repairing an osteochondral defect remains the most challenging part of engineering implants for full thickness osteochondral lesions, which can be repaired through a modular tissue assembly strategy, according to Schon et al. [199].

Furthermore, 3D-printed tissue models may be used to test the efficacy and toxicity of new drug candidates mimicking the native tissue, thus fostering translation of new therapeutic molecules into clinics [157,200]. Compared to other types of 3D in vitro systems [201], 3D bioprinting has numerous advantages such as the controllability, the high-throughput capability, and the generation of drug-delivery vehicles precisely [202]. Indeed, the DVDOD technology delivers droplets to a specific location in a volumetric manner with a high-throughput capability. This technique has been tested to bioprint pre-osteoblast cells with alginate hydrogel into bone damaged tissue, in a minimally invasive manner, showing the formation of functional tissue [177].

Recently, a 3D bioprinted pseudo-bone drug delivery scaffold for simvastatin was generated to promote bone healing. This scaffold displayed matrix strength, matrix resilience, and porous morphology of healthy human bone [203].

In another work, 3D printed PCL/hydrogel composite scaffolds, loaded with bioactive small molecules (i.e., resveratrol and strontium ranelate) able to target bone cells, have been generated and studied to treat craniomaxillofacial defects. The authors implanted the 3D printed scaffolds, with and without small molecules into a rat model with a critical-sized mandibular bone defect, demonstrating that the bone scaffolds, carried with small molecules, showed enhanced angiogenesis, inhibition of osteoclast activities, and stimulation of MSC osteogenic differentiation with consequent in vivo mandibular bone formation eight weeks after implantation [204]. In Table 2, we present some works potentially relevant for their clinical implications, where 3D bioprinting resulted as useful in repairing bone defects.

### 3.5. Bioprinters

Technological advancements and reducing prices of 3D bioprinters available to the final users have sustained the vitality of the field and facilitated the access to a growing numbers of research groups swelling the number of publications in TE, regenerative medicine, and cancer research [155]. The main commercially available bioprinters on the market are compared in Table 3. For the sake of clarity, customized and complex models, which are often the most advanced, have not been discussed here to avoid straying from the focus of this review.

## 4. Conclusions and Remarks

Even though clinical application of bioprinting technology is still in its infancy, the production of entire and functional organs characterized by relevant dimensions is an attractive challenge in TE. As portrayed before, to get closer to this ambitious goal, several aspects should be considered, such as a functional and hierarchical organized vascular network integrated in the system and the incorporation of the various cell types involved in the organ biology [148,208]. Bone may become paradigmatic in this process, as it seems to be more ahead than other tissues in its way toward clinical application. Significant progress has been made in 3D bioprinting for BTE, combining biomaterials, cells, and factor to obtain engineered bone tissue grafts, able to promote bone regeneration. For instance, bioprinted bone was successfully implanted in pre-clinical models [105] and 3D-printed plastic, ceramic, or metallic implants for bone tissue replacement [208] have been successfully transplanted into humans. Finally, a recent work demonstrated a unique case of transplantation of a 3D-printed bio-resorbable airway splint into an infant [209].

The exponential interest in these technologies is leading multidisciplinary teams to develop new bioinks [33] and post-printing procedures. Indeed, thanks to new self-absorbing polymers and the correct incorporation of specific molecules, mechanical, structural, and biocompatibility properties of these materials will be increased to recreate a correct milieu.

The other great technological challenge will be played in the management of post-printing procedures. In fact, more and more companies are developing different types of bioreactor, both in the field of millufluidics and microfluidics. Correct metabolic management and mechanical stimuli of BTE will therefore be possible.

In conclusion, considering the fast evolution of technology, in the next decade it is plausible to expect that volumetric composite tissues with native tissue-like properties will become printable. Indeed, the development of advanced high-resolution bioprinters with multiple modalities and print-heads (such as the newly created ITOP [63]), will lay the foundation for creating complex heterocellular and vascularized tissues. In this regard, the recent development of 4D bioprinting technology [210] could play a key role, since the integration of the concept of time with the 3D bioprinting technology will permit the development of tissues with high levels of complexity and size [123]. This aspect is particularly relevant since natural tissue regeneration is subjected to dynamic modifications of macro-/micro-structures and composition due to different intrinsic and external stimuli. Thus, a sort of maturation and functionalization of the 3D-bioprinted tissue with time is necessary and can be achieved by 4D bioprinting technology [211].

The technological complexity in these fields will make the need for laboratories with extremely multidisciplinary skills increasingly evident. Moreover, standardized regulatory protocols will need to be established, above all considering the even more increasing necessity to translate into clinical practice the use of these TE products.

## Figures and Tables

**Table 1:** Properties affecting shear stress and viability of some common polymers [136].

Polymer	Concentration	Crosslinking Mechanism	Viscosity Range (Pa∙s)
Methacrylated hylaronic acid/methacrylated gelatin	6–12%	Ultraviolet (UV)	0.1–10000
PEG-DA + Laponite	10% PEG-DA, 4% Laponite	UV	1200
Sodium alginate	3–5%	Ionic	0.6–6.4
GelMA	3–5%	UV	75–2000
Hyaluronic Acid	1.5%	Temperature	22
Collagen	1.5–1.75%	Temperature, pH	1.7–1.8

**Table 2:** Applications of 3D Bioprinting on bone defects.

Cell Types, Molecules	Bioink	Bioprinting Modality	Application
Bone marrow MSCs, osteoblast	GelMA + nanocrystalline HA [189]	LBB (Stereolithography)	Breast cancer bone metastases
Osteoblast, breast cancer cells	PEG hydrogel + nanocrystalline HA [205] Hydrogel resins (PEG, PEG-diacryilate) [190]	LBB (Stereolithography)	Breast cancer bone metastases
Without cells	(PLA) and acrylonitrile butadiene styrene (ABS) [204]	EBB with Fused deposition model (FDM)	Radius fracture repair
Periosteal derived cells	Alginate hydrogel + collagen I, II [193,206]	EBB by piston-driven system	Periosteum Tissue Engineering
MSCs	RGD alginate hydrogels [195]	EBB by multiple-head 3D printing system	To engineer endochondral bone
ASCs	HA-GelMA [198,199]	EBB by Biopen	Regeneration of chondral lesions
Meniscal fibrochondrocytes (MFCs)	meniscus extracellular matrix (MECM)-based hydrogel [207]	3D printing fused deposition modeling	Meniscus regeneration
IPS cells, 143B human osteosarcoma cells, preosteoblasts MC3T3	Alginate hydrogel [177]	Direct- volumetric Drop-on-demand (DVDOD) technology	Microtissue fabrication and drug delivery
Simvastatin	copolymeric blend of polymers: polypropylene fumarate (PPF), PEG-PCL-PEG, and pluronic PF 127 [203]	LBB	Drug delivery
Resveratrol and strontium ranelate	PCL/hydrogel [204]	EBB	Cranio-maxillofacial regeneration

**Table 3:** In the table are reported the names with a brief description of the main available bioprinters and their price range (legend: 0–50k$ = $; 50–100k$ = $$; 100–200k$ = $$$; 200–300k$ = $$$$; >300k$ = $$$$$).

Name	Description	Type	Price	Link
**Advanced Solutions** BioAssemblyBot^®^	Robot combining a 3D bioprinter with a robotic arm. The highly maneuverable si*x*-axis robot is capable to hold a variety of interchangeable tools, making the system partly modular. Among the tools one must mention syringe extruders that can be heated/cooled in the range 5–110 °C and a video camera for monitoring the ongoing process. Up to 16 materials may be printed at the given process parameters.	Extrusion-based	$$-$$$$	https://www.advancedsolutions.com/bioassemblybot
**Allevi** Allevi-3^®^	Compact system endowed with three temperature-controlled syringe extruders (4–160 °C) supported by light sources (UV and Visible) for curing/cross-linking printed material. The extrusion pressure up to 120 PSI (allowing a wide range of viscosities) and the calibration is automatic.	Extrusion-based	$	https://www.allevi3d.com/allevi-3
**Aspect Biosystems** RX1^®^	Bioprinter released in 2019. This technology uses microfluidic chips that allow the mixture of materials on-the-fly during printing. The microfluidic channels contain pneumatic valves that allow you to change and mix materials on-the-fly during printing. This capability streamlines the printing process by removing time-consuming steps (i.e., pre-mixing bio-inks; swapping syringes) so print time is only dependent on print volume. On-the-fly mixing paves the way for the RX1′s chemical cross-linking and the formation of cell-laden microfibers using coaxial flow focusing.	Extrusion-based/microfluidic channels	$-$$	https://www.aspectbiosystems.com/technology
**Cellink** Bio X^®^	One of the most user-friendly and flexible bioprinter available. Three print heads may support different print-heads (Heated Pneumatic (rt-65°); Electromagnetic Droplet (rt-65°); Temperature-controlled Pneumatic (4–65°); Syringe Pump (rt-65°); Thermoplastic (250°); Photocuring Toolhead; HD camera). This bioprinter is composed by a little and simple hood with a patented Clean Chamber technology, which uses HEPA filters, UV-C germicidal control (for sterilization cycles) and positive air pressure inside chamber to maintain a pristine workspace. The BioX is equipped with a temperature-controlled printing-bed. A lot of different biomaterials are developed by CELLINK.	Extrusion-based/Ink-jet based	$-$$	https://www.cellink.com/global/bioprinting/bio-x/
**Cellink** LumenX^®^	Light-based bioprinter that offers high resolution, high throughput, and high fidelity—enhancing applications in microfluidics, cell-laden hydrogels, macroporous structures. This bioprinter is designed to bioprint vasculature with biocompatible blue light.	Light-based	$	https://www.cellink.com/bioprinting/lumen-x/
**GeSim** BioScaffolder^®^	Capable of creating bioscaffolds for cell growth or depositing layers of bioinks on implants or microfluidic objects. This bioprinter combines three capabilities: 3D printing, electrospinning, and pipetting. This allows the system to print or electrospin micro-scale fibers, which make up a scaffold, and then pipette small quantities (down to nanoliters) of low-viscosity material onto the scaffold. The pipetted material can be solutions of cells, proteins, or drugs. The system has three extruders for sequential printing of different materials and also includes the latest innovations, namely heating/cooling (0–250 °C), an FDM extruder to print commercial filaments, and coaxial extrusion to form hollow fibers, etc.	Extrusion-based/electrospinning	$$$-$$$$	https://gesim-bioinstruments-microfluidics.com/bioprinter/
**Cyfuse Biomedical** Regenova ^®^	Very useful tool in high-throughput applications. The Regenova system arranges cells (no scaffolds) using micro needle arrays. Cell aggregates (a.k.a. spheroids) are selected, picked up and skewered onto long, 170 micrometrer-wide needles. The system can be automated to select a wide variety of cell types and plant them at specific locations in the array, giving rise to 3D heterogeneous tissues.	Extrusion-based	$$$$-$$$$$	https://www.cyfusebio.com/en/product/3dprinter/device/
**RegenHU** 3DDiscovery Evolution ^®^	Partly modular system composed of a 3D bioprinter and a si*x*-axis robotic arm holding a variety of different tools, including syringe extruders and a video camera. Extruders can be heated/cooled in the range 5–110 °C.	Extrusion-based	$$$$	https://www.regenhu.com/3d-bioprinters
**Rokit’s Healthcare** Dr. Invivo4D^®^	System endowed with a closed chamber equipped with sterilization functionalities. This bioprinter is featuring a temperature control (−10 to 80 °C standard, optional tool goes up to 350 °C). Also available are a wireless control and the possibility for both UV and chemical cross-linking. The technology is based on a dual extruding system.	Extrusion-based	$	http://rokithealthcare.com/invivo/#cd1d104a-bdb2

## Abbreviations

3D	Three-dimensional
ASCs	Adipose-derived stem cells
BMP	Bone morphogenic protein
BMSCs	Bone-marrow-derived human mesenchymal stem cells
BTE	Bone tissue engineering
CAD/CAM	Computer-aided design/computer-aided manufacturing
CaP	Calcium phosphates
CT	Computed Tomography
DBB	Droplet-based bioprinting
DCB	Decellularized bone
dECM	Decellularized extra cellular matrix
DVDOD	direct-volumetric drop-on-demand
ECM	Extra cellular matrix
EBB	Extrusion-based bioprinting
FDM	Fused Deposition -modelling
GelMA	Methacrylated gelatine
HA	Hydroxy-apatite
HUVEC	Human umbilical vein endothelial cells
HyA	Hyaluronic acid
IBB	Inkjet-based bioprinting
iPSCs	induced pluripotent stem cells
ITOP	integrated tissue-organ printer
LBB	Light-based bioprinting
LED	Light-emitting diode
MECM	meniscus extracellular matrix
MSCs	Mesenchimal stem cells
PCL	Polycaprolattone
PDCs	Periosteum tissue by printing periosteal derived cells
PEG	Polyethene glycol
PLA	Poly-lactic acid
PLGA	Poly-lactic-co-glycolic acid
PP	Piperazine
PPF	Polypropylene fumarate
P-PUU	Polyurethane-urea
PU	Thermoresponsive biodegradable polyurethane
PVA	Polyvinyl alcohol
RGD	arginine-glycine-aspartic acid
RM	Regenerative medicine
TCP	Tricalcium phosphate
TE	Tissue Engineering
TGF-β	Transforming growth factor beta
UV	ultraviolet
VEGF	Vascular endothelial growth factor
